# Defense mechanisms of *Salmonella* against antibiotics: a review

**DOI:** 10.3389/frabi.2024.1448796

**Published:** 2024-09-17

**Authors:** Anuradha Jeewantha Punchihewage-Don, Priyanka Nilmini Ranaweera, Salina Parveen

**Affiliations:** ^1^ Department of Agriculture, Food, and Resource Sciences, University of Maryland Eastern Shore, Princess Anne, MD, United States; ^2^ Department of Computer Science and Informatics, Uva Wellassa University, Badulla, Sri Lanka

**Keywords:** *Salmonella* mechanisms, antibiotic resistance, multi-drug resistance, efflux pumps, biofilm formation, plasmid-mediated resistance, antibiotic target modification, drug inactivation

## Abstract

*Salmonella* is a foodborne pathogenic bacterium that causes salmonellosis worldwide. Also, *Salmonella* is considered a serious problem for food safety and public health. Several antimicrobial classes including aminoglycosides, tetracyclines, phenols, and β-Lactams are used to treat *Salmonella* infections. Antibiotics have been prescribed for decades to treat infections caused by bacteria in human and animal healthcare. However, intensive use of antibiotics resulted in antibiotic resistance (AR) among several foodborne bacteria including *Salmonella*. Furthermore, multi-drug resistance (MDR) of *Salmonella* has increased dramatically. In addition to MDR *Salmonella*, extensively drug resistant (XDR) as well as pan drug resistant (PDR) *Salmonella* were reported globally. Therefore, increasing AR is becoming a serious universal public health crisis. *Salmonella* developed many mechanisms to ensure its survival against antimicrobials. The most prominent defense mechanisms against these antibiotics include enzymatic inactivation, expelling drugs from the cell through efflux pumps, altering the structure of drugs, and changing or protecting the targets of drugs. Additionally, the formation of biofilms and plasmid-mediated AR by *Salmonella*, enhancing its resistance to various antibiotics, making it a challenging pathogen in both healthcare and food industry settings. This review focuses exclusively on providing a detailed overview of the mechanisms of AR in *Salmonella*.

## 1 Introduction

*Salmonella* is a member of the Enterobacteriaceae family, a facultative anaerobic, Gram-negative, rod-shaped bacterium that causes the foodborne disease salmonellosis (Andersen et al., 2015; Punchihewage-Don et al., 2022, 2024). The *Salmonella* genus primarily comprises two species: *Salmonella bongori* and *Salmonella enterica*. *S*. *enterica* is categorized into six subspecies including *Salmonella enterica* subsp. *arizonae*, *diarizonae*, *enterica*, *indica, houtenae*, and *salamae*. These subspecies are further divided into serogroups based on the immunoreactivity of cell surface structures, the O (the outermost layer of the bacteria’s surface) and H (thin, thread-like structures found in the flagella) antigens (CDC, 2011, 2022; Ryan et al., 2017; Ferrari et al., 2019; Popa and Papa, 2021). Based on the immunoreactivity of cell surface structures of *Salmonella*, over 2,500 different serovars have been identified to date (WHO, 2018).

Broadly, *Salmonella* can be further categorized into typhoidal and non-typhoidal types. Typhoidal *Salmonella*, which includes *Salmonella enterica* serovar Typhi, Sendai, and Paratyphi A, B, C, are host-adapted to humans and cause systemic and sometimes life-threatening infections such as typhoid and paratyphoid fever, depending on the serovar that invades human cells (Gal-Mor et al., 2014; Smith et al., 2016; Punchihewage-Don et al., 2021; Schultz et al., 2021; Plumb et al., 2023). In contrast, non-typhoidal *Salmonella*, such as *Salmonella enterica* serovar Enteritidis and Typhimurium, typically cause gastroenteritis and can infect a wide range of hosts. Some serovars are host-specific (e.g., *S.* Dublin in cattle and *S*. Choleraesuis in pigs), while many serovars can infect a variety of hosts worldwide, causing disease in both humans and animals (WHO, 2018; CDC, 2022). Non-typhoidal *Salmonella* directly causes mild, severe, or life-threatening foodborne poisoning in humans (WHO, 2018). Notably, *Salmonella* infections are transmitted through a wide range of contaminated products such as raw/uncooked or undercooked meat and poultry or poultry products, fresh fruits and vegetables as well as unpasteurized milk and other dairy products (FDA, 2020; Punchihewage-Don et al., 2021).

The rapid development in antibiotic resistance (AR) among pathogenic *Salmonella* strains in recent years has had a significant impact (Punchihewage-Don et al., 2023). This is because antibiotic-resistant *Salmonella* is directly linked to a rise in human deaths, extended hospital stays, and increased treatment costs due to the failure of therapies (Jajere, 2019). According to the Centers for Disease Control and Prevention (CDC), each year in the United States, *Salmonella* is linked to roughly 1.35 million cases of infection, leads to 26,500 hospital admissions, and results in 420 deaths (CDC, 2023). Furthermore, among these cases, the CDC estimates that about 212,500 are due to drug-resistant non-typhoidal *Salmonella* each year, leading to roughly 70 deaths. Additionally, these infections incur a significant financial burden, amounting to an estimated $400 million in direct medical costs annually (CDC, 2019, 2023). Diarrhea, fever, and stomach cramps are the common symptoms of salmonellosis. Antibiotics are used as a treatment in severe cases of salmonellosis (CDC, 2023). A broad spectrum of antibiotics such as β-lactams, aminoglycosides, tetracyclines, quinolones, cephalosporins, and trimethoprim-sulfamethoxazole are used to combat *Salmonella*. The improper and overuse of antibiotics contributes to the development of AR in pathogenic bacteria (Akinyemi and Ajoseh, 2017; Millan, 2018). Moreover, the reason for the emergence of AR is that bacteria change their response to these antibiotics (WHO, 2023).

Antibiotics help to prevent and fight against infections caused by bacteria such as salmonellosis, pneumonia, tuberculosis, gonorrhea, etc. (WHO, 2023). Overuse of antibiotics has resulted in the development of pathogenic bacteria that are resistant to antibiotics, including multi-drug resistant (MDR) strains. The reasons for AR for not only the overuse and misuse of antibiotics. Several other reasons can confer AR, such as poor infection prevention and control. Individuals, policymakers, health professionals, the healthcare industry, and the agricultural sector are responsible for minimizing the influence and limiting the spread of AR (WHO, 2023). Some strains of *Salmonella* have developed resistance to several classes of antibiotics such as aminoglycosides, β-lactam antibiotics, chloramphenicol, quinolones, tetracyclines, sulphonamides, and trimethoprim (Urban-Chmiel et al., 2022). **Table 1** describes the summary of the *Salmonella* AR mechanisms.

**Table 1:** Brief categorization of clinically important antibiotics, resistance genes, resistance mechanisms and mode of action of *Salmonella*.

Type of antibiotic	Description	Resistance genes	Resistance genes location(s)	Mode of action	Resistance mechanisms	References
Sulfonamides	All the members of this antibiotic class contain the sulfonamide group. Sulfa drugs or sulfonamides were discovered by Gerhard Domagk in 1935.	*sul1* *sul2* *sul3*	Chromosome Plasmid	Sulfonamides inhibit the synthesis of folic acid in bacteria	▪ Sulfonamides antibiotic mainly inhibit dihydropteroate synthase (DHPS). ▪ Dihydropteroate synthase is the crucial enzyme in the folate pathway of bacteria.	(Wang et al., 2022b) (Akinyemi and Ajoseh, 2017) (Jeśman et al., 2011) (Ovung and Bhattacharyya, 2021)
Quinolones	Nalidixic acid was the first introduced drug in the Quinolones drug group by George Lesher and colleagues in 1962. Quinolones are broad-spectrum antibiotics that can combat both Gram-negative and positive bacteria.	*gyrA* *gyrB* *parC* *parE* *qnrB* *qnrD* *qnrS* *oqxAB*	Chromosome Plasmid	Interfere with bacteria DNA replication and transcription	▪ Mutation in the Quinolones Resistance Determining Region (QRDR) *GyrA, GyrB, parC, parE.* ▪ Modification of the target site.	(Wang et al., 2022b) (Akinyemi and Ajoseh, 2017) (Urban-Chmiel et al., 2022) (Cosby et al., 2015) (Aldred et al., 2014) (Pham et al., 2019)
β-Lactams	β-Lactams are a widely used antibiotic group. All the members of the β-Lactam group consist of a β-lactam ring. β-Lactams consist of five relevant ring systems including penem, carbapenem, cefem and monobactam ring structures Penicillin was the first discovered β-Lactams antibiotic by Alexander Fleming in 1928	*bla_TEM_* *bla_TEM−1_* *bla_TEM−20_* *bla_TEM−52_ bla_CTX−M−1_* *bla_CMY−2_* *bla_OXA−1_* *bla _PSE−1_*	Chromosome Plasmid	Inhibit bacteria cell wall synthesis	▪ Preventing the interaction between the target PBP and the drug. ▪ The presence of efflux pumps that can extrude β-lactam drug. ▪ Hydrolysis of the drug by • β-lactamase enzymes	(Mąka and Popowska, 2016) (Wang et al., 2022b) (Akinyemi and Ajoseh, 2017) (Urban-Chmiel et al., 2022) (Reygaert, 2018) (Fernandes et al., 2013) (Lee et al., 2016)
Chloramphenicol	Parke–Davis discovered Chloramphenicol in the late 1940s from the soil bacterium *Streptomyces venezuelae* Chloramphenicol antibiotic has been used to treat several infections such as conjunctivitis, meningitis, cholera, and typhoid fever. Chloramphenicol is active against several bacteria such as *Escherichia coli*, *Staphylococcus* spp., and *Salmonella*.	*catA1* *floR* *cmlA1*	Chromosome Plasmid	Inhibit microbial protein synthesis by binding to the 50S ribosome subunit	▪ Expression of efflux pumps (*floR, cmlA*). ▪ Enzymatic inactivation of the antibiotic by chloramphenicol- col O-acetyl-transferase.	(Akinyemi and Ajoseh, 2017) (Cosby et al., 2015) (American Chemical Society, 2020)
Aminoglycosides^*^	In 1940 Selman Waksman discovered Streptomycin as the first member of the aminoglycoside antibiotic class. This group of antimicrobials is natural or semisynthetic antibiotics that derived from the actinomycetes. This antibiotic class is composed of amino sugars and an aminocyclitol ring	*armA* *rmtC* *aadA1* *aadA2* *aadA5 aphA1AB aac(3)-IV aph(3’)IIa* *aacC2 aac(3)IVa* *aacA4* *strA* *strB* *aadA* *aphA2* *aphA1*	Chromosome Plasmid	Binding to A-site on the 16S ribosomal RNA of the 30S ribosome and block the protein synthesis	▪ Enzymatic modification and inactivation of the aminoglycoside.	(Mąka and Popowska, 2016) (Akinyemi and Ajoseh, 2017) (Kotra et al., 2000) (Krause et al., 2016) (Serio et al., 2018)
Tetracyclines	Tetracycline is the broad-spectrum antibiotic class active against both Gram-negative and positive bacteria. All the members of the tetracyclines antibiotic group have a four-ring structure. Aureomycin (chlortetracycline) was the first recognized antibiotic in this drug class. Terramycin (oxytetracycline) was found by Alexander Finlay and colleagues in 1950. Tetracyclines have three generations respectively first (chlortetracycline, oxytetracycline, demeclocycline) second (doxycycline, lymecycline, meclocycline, methacycline, minocycline, rolitetracycline) and third generations (tigecycline).	*tetA* *tetB* *tetC* *tetD* *tetG*	Plasmid	Block the protein synthesis by binding to the 30S ribosomal subunit	▪ Efflux pumps. ▪ Modification of rRNA target. ▪ Inactivation of compound.	(Mąka and Popowska, 2016) (Akinyemi and Ajoseh, 2017) (Tariq et al., 2018)

^*^According to the Clinical and Laboratory Standards Institute (CLSI) guidelines, aminoglycosides may appear active *in vitro* against *Salmonella*. but are not effective clinically. Therefore, aminoglycosides should not be tested or reported as susceptible for *Salmonella*. in clinical settings (Humphries, 2023).

There are two major molecular mechanisms involved in AR: intrinsic resistance and acquired resistance. Intrinsic resistance, also known as natural or innate resistance, is a natural property of certain bacterial species that makes them resistant to specific antimicrobial agents. This type of resistance is genetically predetermined and persistent, as it is an inherent feature of the organism’s genome (Tanwar et al., 2014; Abebe et al., 2016; Reygaert, 2018). Intrinsic resistance is a universal trait within a bacterial species, independent of previous antibiotic exposure, not related to horizontal gene transfer, and controlled by chromosomal genes (Cox and Wright, 2013). Mechanisms of intrinsic resistance include producing enzymes that neutralize antimicrobial compounds, structural barriers that prevent compounds from reaching target sites, reduced permeability of the bacterial outer membrane, natural activity of efflux pumps, and the lack of appropriate target sites for antibiotics. Bacteria with intrinsic resistance often employ these mechanisms to withstand the effects of antimicrobial agents (Cox and Wright, 2013; Olivares Pacheco et al., 2013; Abebe et al., 2016; Reygaert, 2018).

In contrast, acquired resistance refers to the ability of bacteria to become resistant to antibiotics through genetic changes. This type of resistance develops in previously susceptible bacteria and can occur through genetic mutations or acquisition of resistance genes from other resistant bacteria (Reygaert, 2018; Mancuso et al., 2021; Chin et al., 2023; Harris et al., 2023). Mechanisms of acquired resistance include modification of antibiotic target sites, production of enzymes that inactivate antibiotics, and enhanced efflux of antibiotics from bacterial cells. Acquired resistance can spread through horizontal gene transfer involving processes like transformation, transposition, and conjugation (Culyba et al., 2015; Abebe et al., 2016; Reygaert, 2018; Mancuso et al., 2021; Harris et al., 2023). Both single-drug resistant and MDR pathogenic microorganisms developed resistance mechanisms as their surveillance strategy (Andersen et al., 2015).

Notably, over the years, most *Salmonella* have evolved resistance mechanisms to numerous antibiotics (Akinyemi and Ajoseh, 2017; CDC, 2019). As a result, AR in *Salmonella* is a rising problem in the food industry as well as the health sector (Mąka and Popowska, 2016; Punchihewage-Don et al., 2022, 2024). AR has been identified in both typhoidal and non-typhoidal *Salmonella* serovars (Baugh, 2014; CDC, 2019; Punchihewage-Don et al., 2024). Moreover, MDR *Salmonella* increased its prevalence by combating clinically essential antibiotics such as fluoroquinolones and third-generation cephalosporins (CDC, 2019; Jajere, 2019; Punchihewage-Don et al., 2022). Several AR mechanisms are encoded in the genome of *Salmonella* such as active drug efflux pumps, decreased membrane permeability, enzymatic inactivation of the antibiotics, target site modification, plasmid-mediated resistance, and biofilm formation. These mechanisms allow *Salmonella* cells to expel antibiotics and reduce the antibiotic concentration within the cytoplasm. In addition, these mechanisms are capable of conferring MDR (Shrestha et al., 2015).

Despite extensive knowledge of AR mechanisms in bacteria, a significant gap exists in understanding emerging resistance patterns and the role of novel efflux pump families like PACE and AbgT in *Salmonella*. This review addresses this gap by analyzing various AR mechanisms, including these new efflux pumps. By synthesizing recent findings, we aim to provide a detailed overview of the AR mechanisms exclusively for *Salmonella*.

## 2 Drug efflux

**Figure 1:**
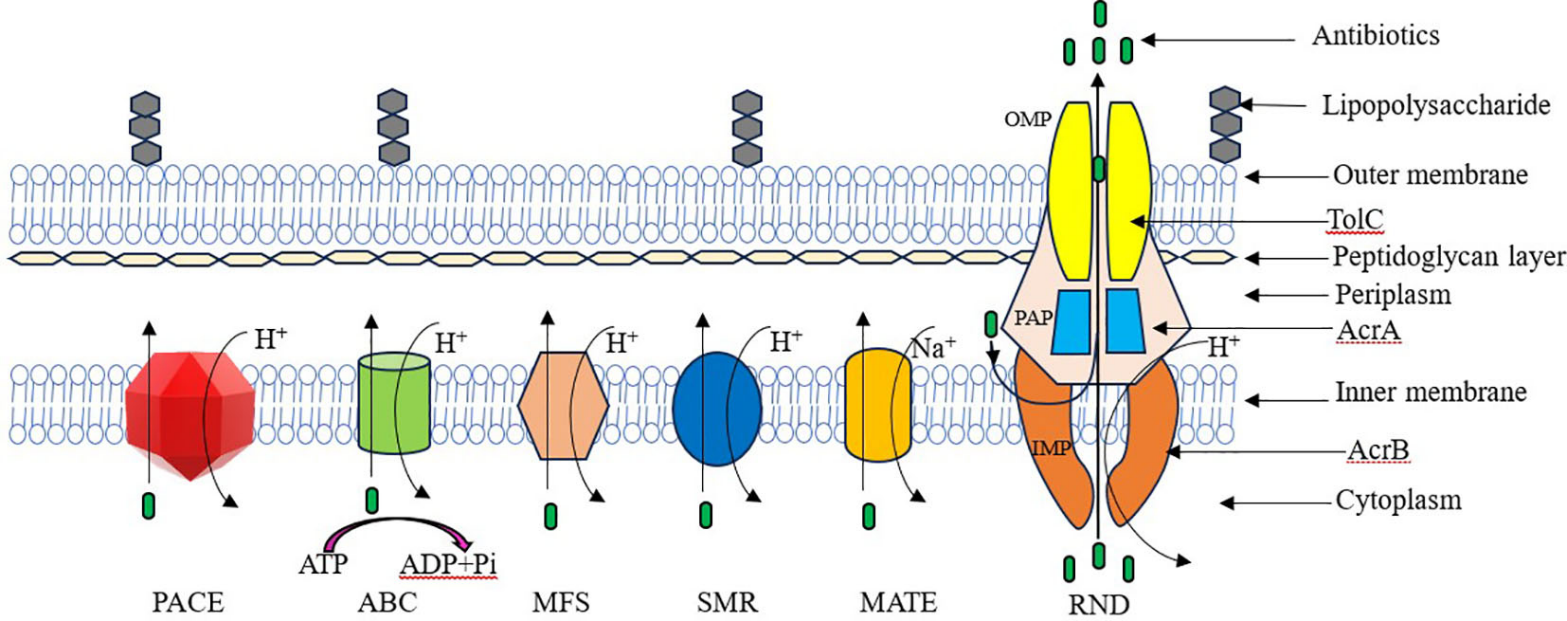
The general structure of main efflux families.

Antibiotic efflux is a widely used AR mechanism among bacteria. This mechanism allows bacteria to extrude antibiotics from their intracellular environment to the extracellular environment (Noto, 2022). *Salmonella* utilizes different types of efflux pumps. Those drug efflux systems are divided into five main families depending on their energy source and structure (**Figure 1**). They are ATP-binding cassette (ABC) family, small multi-drug resistance (SMR) family, multi-drug and toxic compound extrusion (MATE) family, major facilitator superfamily (MFS), and resistance nodulation cell division (RND) family (Reygaert, 2018; Nishino et al., 2021; Gaurav et al., 2023; Duffey et al., 2024).

Efflux pumps can be categorized by their energy sources into primary and secondary active transporters. Primary active transporters, such as members of the ABC family, obtain energy through the hydrolysis of ATP. In contrast, secondary active transporters, including members of the MATE, MFS, RND, and SMR families, utilize energy derived from hydrogen ions (H^+^) or the electrochemical gradient of sodium ions (Na^+^), generated by the proton motive force (Sharma et al., 2019).

*Salmonella* encodes at least eleven MDR pumps from each family except the SMR family (Bogomolnaya et al., 2013). *S*. Typhimurium harbor functional drug efflux pump systems that belong to four main efflux pump system families. These efflux pump systems belong to the following families: the MFS family (including EmrAB and MdfA), the ABC family (MacAB), the MATE family (MdtK), and the RND family (AcrAB, AcrD, AcrEF, MdtABC, MdsAB) (Andersen et al., 2015). According to the latest studies, two new bacterial energy efflux pump families have been discovered. These novel drug efflux systems are the proteobacterial antimicrobial compound efflux (PACE) superfamily and the p-aminobenzoyl-glutamate transporter (AbgT) family (Hassan et al., 2018).

### 2.1 Resistance nodulation cell division family

Resistance nodulation cell division (RND) family effluxes are more effective for the extrusion of a wide range of antibiotics such as chloramphenicol, novobiocin, tetracyclines, some β-lactams, fusidic acid and fluoroquinolones, detergents, bile salts, metals and biocides (Alenazy, 2022b). The RND family is crucial to MDR in *Salmonella* (Shrestha et al., 2015). The three major proteins are involved in forming the tripartite efflux system in RND family efflux pumps. They are an inner-membrane protein (IMP), an outer-membrane protein (OMP), and a periplasmic adapter protein (PAP) (Alenazy, 2022b). There are five RND multi-drug efflux systems belonging to *Salmonella*. They are AcrAB, AcrD, AcrEF, MdtABC, and MdsABC efflux pumps (Yamasaki et al., 2011). The AcrAB-TolC efflux system is the common and extensively studied drug efflux pump system in *Salmonella*. The AcrAB drug efflux pump system of *S*. Typhimurium is located on the chromosome and it is encoded by the *acrAB* genes. The AcrAB-TolC system comprises a tripartite efflux pump in *Salmonella*. Furthermore, the AcrAB-TolC efflux pump forms three protein subunits. They are AcrB (inner membrane transporter protein), AcrA (periplasmic adaptor protein), and TolC (the outer membrane porin protein) (Baugh, 2014).

The tripartite AcrAB-TolC drug efflux pump system of *S*. Typhimurium vigorously excludes a wide range of antibiotics such as chloramphenicol, tetracycline, ciprofloxacin, acriflavine, fusidic acid, novobiocin, erythromycin, rifampin, and β-lactam. Fernandes et al. (2013) elaborated on the significance of the *Salmonella* AcrAB-TolC system in the pathogenesis of the bacterium (Fernandes et al., 2013). The AcrD efflux pump of *Salmonella* actively extrudes kanamycin, tobramycin, gentamicin, and amikacin. The AcrEF efflux system in *Salmonella* is homologous and functionally similar to the AcrAB system, and together with TolC, forms the AcrEF-TolC tripartite multi-drug efflux system that complements the AcrAB-TolC system’s function. In *Salmonella*, the AcrEF-TolC tripartite multi-drug efflux system is composed of three components. They are AcrE (membrane fusion protein), AcrF (inner membrane transporter), and TolC (outer membrane channel protein). The member of AcrEF efflux in family RND resistance to ciprofloxacin, tetracycline, nalidixic acid, chloramphenicol, and triclosan. The MdsABC efflux pump is found only in *Salmonella* (Shrestha et al., 2015).

Inner membrane RND-type transporter (MdsB), periplasmic membrane-fusion protein (MdsA), and outer membrane protein (MdsC) are the major three components of the *Salmonella* MdsABC tripartite efflux pump (Song et al., 2015). *Salmonella* MdsABC efflux pump resistant to novobiocin, deoxycholate, some β-lactams, copper and zinc (Blair et al., 2014). MdtABC drug efflux systems of *Salmonella* have two different transporters called MdtB and MdtC. Both of them are co-transcribed along with MdtA (a membrane fusion protein) in the same operon. In *Salmonella*, MdtABC efflux pumps resistance to antibiotics such as β-lactams, novobiocin, and bile salts. Furthermore, the MdtABC efflux pump detoxified the cell from copper, zinc, and tungstate (Shrestha et al., 2015).

### 2.2 Small multi-drug resistance family

The small multi-drug resistance (SMR) family is the smallest efflux transporter family among the other four families. This family of efflux transporters is restricted to prokaryotic cells (Higgins, 2007). The SMR family obtains energy from the proton-motive force (H^+^) (Reygaert, 2018). SMR transporters confer resistance to a diverse number of quaternary ammonium compounds and lipophilic cations like benzalkonium, cetyltrimethylammonium bromide (CTAB), cetylpyridinium chloride (CTPC), methyl viologen, and tetraphenylphosphonium (TPP). In addition, SMR efflux pumps confer resistance to antibiotics such as β-lactams, cephalosporins, dihydrofolate inhibitors, and aminoglycosides (Shrestha et al., 2015).

### 2.3 ATP-binding cassette family

The ATP-binding cassette (ABC) family of drug efflux pumps derives its energy from ATP hydrolysis to remove drugs from the intracellular environment (Kabra et al., 2019). The ABC family is known as the primary active transporters because it utilizes ATP as its energy source to extrude substances out of the cell. MacAB-TolC is one of the known drug efflux pump systems in *Salmonella.* MacAB-TolC can form tripartite complex. MacB functions as the inner membrane pump protein (efflux pump). Periplasmic protein and the outer membrane channel of the *Salmonella* MacAB-TolC tripartite efflux pump are respectively MacA and TolC. In *Salmonella*, MacAB-TolC Efflux system resistance to the macrolides such as erythromycin, and azithromycin (Baugh, 2014). In *Salmonella*, the PhoPQ regulatory system, which is a two-component system, controls the expression of the MacAB pump (Nishino et al., 2006, 2009). Specifically, PhoP, a component of the PhoPQ system, binds to the macAB ABC transporter and represses its activity (Nishino et al., 2006). This regulatory mechanism is crucial in controlling the virulence of *Salmonella* (Kato and Groisman, 2008). Furthermore, the functions of the PhoPQ system in *Salmonella* are multifaceted, encompassing various key physiological processes. These include the regulation of Mg^2+^ homeostasis, providing resistance against antimicrobial peptides, and enabling growth in acidic environments with a low pH level (Groisman et al., 2021). Moreover, the MacAB drug efflux system, a member of the ABC family in *Salmonella*, serves as a critical defense mechanism, safeguarding the bacteria from oxidative stress. This efflux pump is notably activated in response to exposure to hydrogen peroxide (H_2_O_2_). Such induction facilitates the survival of *Salmonella* under conditions where peroxide is present, highlighting the pump’s crucial role in the bacterial defense mechanism against oxidative damage (Bogomolnaya et al., 2013).

### 2.4 Major facilitator superfamily

The major facilitator superfamily (MFS) is generally introduced as the largest family of secondary active solute transporters (Kumar et al., 2013). MFS transporters have broad substrate specificity, such as ions, amino acids, carbohydrates, lipids, nucleosides, and other small molecules (Madej, 2014). The EmrAB efflux pump, a member of the MFS family, significantly contributes to the internal drug resistance of *Salmonella.* EmrAB and MdfA are the most described efflux pumps in *Salmonella*. The EmrAB efflux pump comprises the membrane fusion protein EmrA and functions as a multi-drug efflux system, expelling substrates such as novobiocin, nalidixic acid, and sodium deoxycholate from the bacterial cell. The MdfA efflux pump in *S*. Typhimurium acts as a single cytoplasmic efflux protein, conferring resistance to norfloxacin, chloramphenicol, tetracycline, and doxorubicin (Baugh, 2014; Andersen et al., 2015).

### 2.5 Multi-drug and toxic compound extrusion

The multi-drug and toxic compound extrusion (MATE) efflux family acquires energy by using the Na^+^ gradient (Reygaert, 2018). The MdtK efflux pump is a member of the MATE-type drug efflux pump in *Salmonella*. MdtK efflux pump is substrate-specific and excludes antibiotics such as fluoroquinolones, cation drugs, and aminoglycosides from the cells (Alenazy, 2022a). MdtK efflux pump of *Salmonella* confers resistance to antibiotics such as norfloxacin, doxorubicin, and acriflavine (Shrestha et al., 2015).

### 2.6 Proteobacterial antimicrobial compound efflux superfamily

Recently identified, the proteobacterial antimicrobial compound efflux (PACE) superfamily with AceI from *Acinetobacter baumannii* as the prototype. The PACE bacterial drug efflux transport proteins (AceI) are encoded by genes that are discovered in the genomes of numerous pathogenic bacteria species such as *Pseudomonas*, *Klebsiella*, *Enterobacter*, *Salmonella*, and *Burkholderia* species (Huang et al., 2022). The PACE multi-drug transporters are secondary active transporters that utilize the proton motive force to transport biocides such as benzalkonium, diqualinium, acriflavin, proflavin, and chlorhexidine (Hassan et al., 2018; Zhao et al., 2022; Gaurav et al., 2023; Ahmad et al., 2024). PACE family members are particularly common among proteobacteria. There are no findings so far that PACE efflux systems exist in archaea and eukaryotes (Zhao et al., 2022).

### 2.7 p-Aminobenzoyl-glutamate transporter family

The AbgT family of transporters is crucial for bacterial folate synthesis, essential for growth, and acts as a drug efflux pump, facilitating resistance to various sulfa drugs. The AbgT family uses the proton motive force for transporting biocides (Delmar and Yu, 2016). This family includes two primary members, YdaH and MtrF, who play key roles in these biological processes (Shome et al., 2021). Both efflux systems YdaH and MtrF vigorously mediate the bacterial resistance against sulfonamide antimetabolite drugs. AbgT‐type efflux proteins have been discovered in both Gram-negative bacteria (*S. enterica, E. coli*, *N. gonorrhoeae, A. borkumensis)* and Gram-positive bacteria including *Staphylococcus aureus* and *Streptomyces coelicolor.* Also, these efflux proteins can be found in eukaryotes including the yeast *Saccharomyces arboricola* (Delmar and Yu, 2016). However, further research is necessary to understand the prevalence and specific functions of the AbgT transporter family in *Salmonella*. This knowledge gap highlights the need for more in-depth studies to elucidate how these transporters operate and contribute to the bacterial life cycle and drug resistance mechanisms in *Salmonella*.

**Table 2** provides a comparison of the newly discovered PACE and AbgT efflux pump families with the established efflux pump families (ABC, SMR, MATE, MFS, and RND), highlighting differences in energy utilization, substrate specificity, and structural properties.

**Table 2:** Comparison of efflux pump families in *Salmonella*: energy utilization, substrate specificity, and structural properties.

Efflux pump family	Energy utilization	Substrate specificity	Structural properties	References
PACE	Proton motive force	Actively transporting a wide range of structurally diverse antimicrobial compounds including chlorhexidine, benzalkonium, dequalinium, proflavine, and acriflavine	PACE family contains four transmembrane-spanning helices	(Ahmad et al., 2018; Klenotic et al., 2021; Zhao et al., 2022; Gaurav et al., 2023; Ahmad et al., 2024)
AbgT	Proton motive force	Specific for sulfonamide antimetabolite drugs	AbgT transporters contain nine transmembrane-spanning helices	(Delmar and Yu, 2016; Klenotic et al., 2021; Ahmad et al., 2024)
ABC	ATP hydrolysis	Broad range of solutes including drugs, lipids, and sterols	ABC transporters share a common core structure consisting of two transmembrane domains and two nucleotide-binding domains	(Ahmad et al., 2018; Kabra et al., 2019; Klenotic et al., 2021; Gaurav et al., 2023; Zack et al., 2024)
SMR	Proton motive force	Diverse number of quaternary ammonium compounds, lipophilic cations like benzalkonium, cetyltrimethylammonium bromide, cetylpyridinium chloride, methyl viologen, and tetraphenylphosphonium	SMR proteins have four transmembrane-spanning helices	(Shrestha et al., 2015; Ahmad et al., 2018; Reygaert, 2018; Kornelsen and Kumar, 2021; Gaurav et al., 2023; Ahmad et al., 2024; Zack et al., 2024)
MATE	Sodium ion gradient or proton motive force	Substrate-specific and excludes antibiotics including fluoroquinolones, cation drugs, and aminoglycosides	Containing twelve transmembrane spanning helices	(Lu et al., 2013; Alenazy, 2022a; Gaurav et al., 2023; Ahmad et al., 2024)
MFS	Proton motive force	Broad substrate specificity, including ions, amino acids, carbohydrates, lipids, nucleosides, and other small molecules	Most of these family members function as monomeric units and contain 12 to 14 transmembrane helices arranged into two domains, each forming a bundle of six helices	(Madej, 2014; Ahmad et al., 2018; Gaurav et al., 2023; Ahmad et al., 2024)
RND	Proton motive force	Broad spectrum including chloramphenicol, novobiocin, tetracyclines, some β-lactams, fusidic acid fluoroquinolones, detergents, bile salts, metals and biocides	The tripartite complex spans both the inner and outer membranes and usually has twelve transmembrane helices	(Alenazy, 2022b; Gaurav et al., 2023; Ahmad et al., 2024)

## 3 Altered membrane permeability

Reduced permeability is a well-known AR mechanism among *Salmonella* (Akinyemi and Ajoseh, 2017). There are two mechanisms involved in reducing antibiotic permeability in Gram-negative bacteria including *Salmonella*. They are alterations of the outer membrane lipid barrier and porin-mediated permeability. Structural and functional arrangements of *Salmonella*’s lipopolysaccharide (LPS) layer provide a barrier to some molecules including the antibiotics (Sirisena, 2000; Reygaert, 2018). Furthermore, having a hydrophobic lipid bilayer with pore-forming proteins of specific size-exclusion properties leads the external membrane to act as a penetration barrier (Delcour, 2009).

The cell wall of *Salmonella* is mainly composed of three different layers. They are the outer membrane, the peptidoglycan layer, and the inner membrane (Rycroft, 2000). The external membrane of *Salmonella* is an asymmetrical, highly complex lipid bilayer. Its inner layer forms the phospholipids and its outer layer forms the lipopolysaccharides. The lipopolysaccharide molecule is amphipathic. It includes both hydrophobic and hydrophilic components on the same molecule. The LPS molecules consist of three regions: a hydrophobic anchor lipid A, a core oligosaccharide with several anionic moieties, and the O-antigen (Rycroft, 2000). *Salmonella* modifies its own LPS as a mechanism to resist antimicrobial agents such as antimicrobial peptides. PhoPQ and PmrA/B are two pairs of component systems that allow modifications in the LPS of *Salmonella*. The genes crucial for LPS modification in *Salmonella*, such as *pmrD*, *pmrC*, *pmrG*, the *pmrH-M* operon, and *pmrE*, fall under the regulatory control of the two regulatory component systems PhoPQ and PmrAB (Devi, 2011).

In Gram-negative bacteria, the phosphorylation of lipid A and core sugars contributes to the anionic properties of the cell surface. As a strategy, Gram-negative bacteria like *Salmonella* are used to reduce the negative charge of the cell exterior surface through the addition of positive charges into lipid A. This process manipulates the decreasing affinity for antimicrobial peptides. The lipid A segment of LPS undergoes three structural modifications, such as the addition of palmitate, the addition of phosphoethanolamine, and the attachment of aminoarabinose (Abdi et al., 2019). For instance, in *S. typhimurium*, the addition of the amine-containing sugar aminoarabinose to the lipid A phosphate group confers resistance to polymyxin B (Band and Weiss, 2015).

Lipid A acylation is a crucial mechanism by which *Salmonella* resists antimicrobial peptides. This process enhances the bacterium’s ability to reduce the fluidity of its outer membrane by increasing its hydrophobicity. In *Salmonella*, palmitate chains are added to lipid A to form a hepta-acylated lipid A, a process facilitated by the outer membrane enzyme PagP, which is regulated by the PhoPQ system. The hepta-acylated structure of lipid A is significant because it inhibits the incorporation of cationic antimicrobial peptides, thereby protecting the bacteria from these antimicrobial agents (Li et al., 2013; Boll et al., 2015; Matamouros and Miller, 2015; Saha et al., 2022). In addition, a study revealed that *Salmonella* lipopolysaccharide modifications prevent the outer membrane penetration of novobiocin (Nobre et al., 2015).

Porins are protein channels that reside in the outer membrane of Gram-negative bacteria, such as *Escherichia coli* and *Salmonella*. These channels are crucial for the passive transport of small molecules, including nutrients and certain antibiotics, across the outer membrane (Galdiero et al., 2012; Choi and Lee, 2019; Ude et al., 2021). Generally, porins permit the passive transport of hydrophilic molecules with a molecular weight of less than 600 Da. In addition, bacterial porins facilitate the entry of nutrients and the excretion of waste and toxic compounds from the bacterial cell environment. The major porins of *S.* Typhimurium are OmpF, OmpC, PhoE and OmpD (Ipinza et al., 2014). Two major porin-based mechanisms can be identified in Gram-negative bacteria including *Salmonella*, *E. coli*, *P. aeruginosa*, *Klebsiella pneumoniae.* The two mechanisms involve modifications to the outer membrane porins, encompassing their loss, reduction, or alteration, along with specific mutations that directly lead to changes in porin functionality (Andersen et al., 2015). Members of the family Enterobacteriaceae are well-known examples of reducing the number of porins as their resistance mechanism against antibiotics. Sometimes, bacteria completely stop producing certain porins (Reygaert, 2018). In *Salmonella*, OmpC and OmpF porins allow β-lactams to enter the bacterial cell and reach their penicillin-binding protein targets. It has been reported that a reduction in either OmpF or OmpC porin amount leads to an increase in the resistance to β-lactams including ampicillin, cefoxitin, and other cephalosporins (Cosby et al., 2015). In addition, the deficiency of OmpC porin in *S.* Typhimurium can lead to carbapenem resistance (Vergalli et al., 2020). Moreover, a deficiency of OmpF porin expression has been reported in some quinolone-resistant *Salmonella* strains (Al Gallas and Aissa, 2017).

## 4 Inactivation of drugs

Drug inactivation is one of the well-studied antibiotic defensive mechanisms. This mechanism enables the destruction or inactivation of the antibiotic compounds by enzymatic hydrolysis. The β-lactamase enzyme is the most typical example of an enzymatic hydrolysis process (Noto, 2022). Enterobacteriaceae family including *Salmonella* attain resistance to β-lactam by synthesizing β-lactamase or extended-spectrum β-lactamase (Akinyemi and Ajoseh, 2017). The main mechanism of the β-lactamases enzyme is to inactivate the β-lactam antibiotic by hydrolyzing a specific site in the β-lactam ring structure. This process leads to cleaves the β-lactam ring. Furthermore, the open-ring structure of antibiotics is unable to bind their target penicillin-binding protein (PBP) (Reygaert, 2018; Tooke et al., 2019).

Extended-spectrum β-lactamases (ESBLs) are enzymes that confer broad-spectrum AR. Many of these ESBLs are plasmid-mediated enzymes and help bacteria resist a wide range of β-lactams (Bradford, 2001; Naas et al., 2008). ESBLs have been predominantly detected in the Enterobacteriaceae family including *Klebsiella*, *Escherichia coli, Salmonella, Enterobacter*, *Proteus*, *Citrobacter, Serratia* and *Providencia* (Teklu et al., 2019). ESBLs can inactivate broad-spectrum cephalosporins, penicillins, and aztreonam. Additionally, β-lactamase inhibitors such as sulbactam, clavulanic acid, and tazobactam are highly effective against ESBLs (Rahman et al., 2018). *Salmonella* uses enzymatic inactivation mechanisms to resist chloramphenicol and aminoglycosides. Plasmid-mediated resistance genes in the *Salmonella* genome lead to the production of enzymes such as phosphotransferases and chloramphenicol acetyltransferases, which inactivate chloramphenicol (Guerra et al., 2000). Moreover, *Salmonella* inactivates aminoglycosides through the production of aminoglycoside-modifying enzymes (AME) such as adenyltransferases, acetyltransferases, and phosphotransferases (Noto, 2022; Thacharodi and Lamont, 2022).

## 5 Antibiotic target site modification

In this AR mechanism, bacteria can hinder the effectiveness of antibiotics by obstructing their binding sites. Bacteria employ several strategies to change the target sites such as target protection, point mutations in the genes coding the binding site, and replacement or avoidance of the original binding sites (Munita and Arias, 2016; Schaenzer and Wright, 2020). The mutations that occurred in the DNA gyrase and topoisomerase IV are the best examples to discuss under this topic. *Salmonella* develops resistance to fluoroquinolones and quinolones through mutations in DNA gyrase and topoisomerase IV, resulting in structural changes to these enzymes. Also, it allows for the reduction or complete elimination of the antibiotic’s ability to attach to these target sites (Reygaert, 2018; Herrera-Sánchez et al., 2021). DNA gyrase is identified as the primary target of quinolones in salmonellae (Kongsoi et al., 2015). Both enzymes are large and complex, each consisting of two subunit pairs: GyrA (a 97 kDa protein encoded by the gyrA gene) and GyrB (a 90 kDa protein encoded by the gyrB gene). ParC (75 kDa) and ParE (70 kDa) are named as the corresponding subunits of topoisomerase IV. DNA gyrase has the ability to add negative supercoils into DNA molecules and can also abolish both positive and negative supercoils. Additionally, it can catenate and decatenate closed circular molecules. DNA topoisomerase IV can eliminate both positive and negative supercoils as well but plays a better role in decatenation compared to DNA gyrase. Both enzymes collaborate in the replication, transcription, recombination, and repair of DNA (Jacoby, 2005).

Quinolones target the bacterial enzymes DNA gyrase and topoisomerase IV, which are essential for DNA replication. Quinolones inhibit the enzymes’ ability to re-ligate the DNA strands after inducing double-strand breaks, ultimately leading to the accumulation of DNA breaks and bacterial cell death (Drlica and Zhao, 1997; Fàbrega et al., 2009; Aldred et al., 2014; Kongsoi et al., 2015; Hooper and Jacoby, 2016; Pham et al., 2019). Quinolone resistance initiates with the chromosomal mutations in Quinolone Resistance Determinant Regions (QRDR) of DNA gyrase and topoisomerase IV (Li et al., 2018). Furthermore, the major mechanism of *Salmonella* resistance to quinolones is mutations that change the target site of the antibiotics with DNA gyrase. In *Salmonella*, mutations occurred in the specific region (QRDR) of the *gyr*A gene, i.e., between amino acids 67 and 106 (Souza et al., 2011). In *Salmonella*, point mutations in the QRDR of the *gyr*A gene could be adequate to mediate resistance to non-fluorine quinolones such as nalidixic acid and decrease sensitivity to ciprofloxacin. Therefore, additional mutations are needed for a higher level of fluoroquinolone resistance (Souza et al., 2011; Li et al., 2018). Mutation sites within the QRDRs of the *gyrA* gene are positioned at amino acid positions Serine-83 (Ser-83) and Aspartate-87 (Asp-87). The most common amino acid substitutions in nalidixic acid-resistant strains are Leu, Thr, Phe, Tyr, or Ala at Ser-83, and Gly, Lys, Asn, or Tyr at Asp-87. In cases of high-level resistance, double mutations at both positions 83 and 87 in the *gyr*A gene have been identified in clinical isolates of *S*. Typhimurium. These mutations are known to significantly alter the DNA gyrase enzyme, leading to reduced binding of fluoroquinolone antibiotics and thus contributing to high-level resistance. Additionally, *Salmonella* strains can also exhibit amino acid substitution mutations at other positions, such as Ala-67 (to Pro), Gly-81 (to Ser, Asp, Cys, or His), and Leu-98 (to Val) (Li et al., 2018).

Souza et al. (2011) reported that 105 *Salmonella* strains (94 epidemic and 11 of poultry origin) were resistant to nalidixic acid out of a total of 123 *Salmonella* strains (Souza et al., 2011). They aimed to evaluate mutations in the QRDR of the *gyr*A gene related to resistance to the nalidixic acid and decreased susceptibility to ciprofloxacin using allele-specific PCR and restriction fragment length polymorphism (AS-PCR-RFLP). The study found a high incidence of mutations, particularly at codons that code for Asp-87 and Ser-83, which are associated with quinolone resistance. The research concluded that a need for judicious use of quinolones to treat *Salmonella* infections due to the potential for resistance development. Diverse mutations have also been discovered in the QRDRs of DNA gyrase (GyrA and GyrB) and topoisomerase IV (ParC and ParE) of typhoidal *Salmonella*. These mutations are mentioned in **Table 3** (Shaheen et al., 2021). A recent study has identified novel mutations within the QRDR of DNA gyrase and topoisomerase IV in *Salmonella* isolates from Jiangsu Province, China. These findings reveal new insights into the mechanisms of quinolone resistance in *Salmonella*. This research reported seven novel mutations in the GyrB (S426G), ParC (D79G), and ParE (S498T, E543K, V560G, I444S, Y434S) expanding our understanding of the genetic diversity underlying quinolone resistance (Qian et al., 2020). Research on chromosomal alterations in the QRDRs of DNA gyrase and topoisomerase IV in *Salmonella* has provided valuable insights into the emergence and dissemination of quinolone resistance. By identifying specific mutation patterns and understanding their impact on resistance, these studies contribute to improved diagnostic methods, treatment approaches, and surveillance efforts. Ultimately, this knowledge supports the development of targeted strategies to combat quinolone-resistant *Salmonella.*

**Table 3:** Summary of mutations in quinolone resistance determining regions of *Salmonella*.

DNA gyrase		Topoisomerase IV	
*GyrA*	*GyrB*	*ParC*	*ParE*
Arg47Ser	Gly435Glu/Ala/Val	Thr57Ser	Glu420Asn
Asp82Asn	Ser464Tyr/Phe/Thr	Gly72Ser	Tyr434Ser
Ser83Phe/Tyr/Leu	Gln465Leu	Asp79Gly	Ile444Ser
Asp87Asn/Gly/Tyr/Val	Glu466Asp	Ser80Ile	Ser493Phe
Glu133Gly	Ala468Glu	Glu84Lys	Ser498Thr
Asp147Gly	Ala574Val	Glu92Lys	Glu543Lys
	Ser426Gly	Trp106Gly	Val560Gly

## 6 Plasmid-mediated resistance

In this mechanism, bacteria transfer AR genes which are carried on plasmids (Millan, 2018). Plasmids are small, extrachromosomal circular DNA molecules primarily found in bacteria, but they also exist in some eukaryotes. Plasmids replicate independently, and the genes they carry provide genetic advantages to bacteria, such as AR. Furthermore, plasmids facilitate the horizontal transfer of resistance genes between pathogenic bacteria through conjugation (Bennett, 2008; National Human Genome Research Institute, 2014).

Resistance plasmids contain one or more AR genes, and the majority of them have the ability to transfer between bacteria through conjugation (Bennett, 2008). AR plasmids comprise single or multiple AR genes that can develop an MDR phenotype (Nikaido, 2009). Plasmids carry AR genes in bacteria, conferring resistance to various antibiotics. These plasmids, which possessed AR genes, are known as R factors or resistance plasmids. These R factors are categorized into specific groups known as incompatibility groups (Inc groups) (Rycroft, 2000). Plasmid incompatibility refers to the inability of two plasmids to persist simultaneously within the same cell line (Singh et al., 2022). Plasmids from various incompatibility groups have been connected to various AR genes in *Salmonella* and other bacteria (Carattoli, 2003). Specifically, IncA/C plasmids isolated from *Salmonella* carry genes that exhibit resistance to various antibiotics, including aminoglycosides, β-lactams, chloramphenicol, sulfisoxazole, tetracyclines, and trimethoprim (McMillan et al., 2019).

Most AR genes in *Salmonella* are frequently found on plasmids. *Salmonella* contains multiple large conjugative plasmids carrying AR genes, conferring resistance to antibiotics such as β-lactams, tetracyclines, aminoglycosides, and quinolones (Millan, 2018). The conjugation of plasmids carrying AR mechanisms has contributed to the universal dissemination of AR genes within the Enterobacteriaceae family, including *Salmonella*. These plasmids have the capacity to confer resistance to various antibiotics, including β-lactamases (CMY, DHA, GES, LAP, NDM, SHV, TEM), extended-spectrum β-lactamases (CTX, VEB), metallo β-lactamases (IMP), carbapenemases (KPC, VIM), quinolone resistance (Qep, Qnr), aminoglycoside resistance (AAC, Arm, RmtB), tetracycline resistance (Tet), sulfonamide resistance (Sul), and colistin resistance (Huddleston, 2014).

The resistance genes like *bla*_SHV_, *bla*_TEM_, *bla*_CTX_, *bla*_CMY,_ and *bla*_OXA_ encoded by *Salmonella* plasmids are responsible for the ESBL plasmid-mediated resistance (Mąka and Popowska, 2016). *Salmonella* primarily employs an enzymatic inactivation mechanism as its main defense strategy to resist ESBLs. Furthermore, plasmid-mediated resistance has played a significant role in conferring resistance to ESBLs among *Salmonella*. These plasmids often carry genes like *bla_CMY-2_* and *bla_CTX-M-3_*, which are responsible for resistance and can be shared among bacterial organisms, regardless of bacterial species. This has contributed to the widespread occurrence of ESBL resistance (Su and Chiu, 2007).

Quinolones are widely used to treat salmonellosis in both human and veterinary medicine (Pribul et al., 2017). However, their continuous application over the years has resulted in an increase in resistance (Jacoby et al., 2014). Bacteria exhibit three main mechanisms to resist quinolones: protection of the target from the antibiotics through plasmid-mediated genes, modification of antibiotics, and reducing intracytoplasmic quinolone concentration through efflux pumps (Jacoby et al., 2014; Ruiz, 2019; Usman and Hamza, 2022). Notably, the main three defensive mechanisms for Plasmid-Mediated Quinolone Resistance (PMQR) have been revealed since 1988 (Jacoby et al., 2014; Ruiz, 2019). In brief, PMQR involves genes such as *qnrA*, *qnrB*, *qnrC*, *qnrD*, *qnrS*, and *qnrVC*, which produce proteins from the pentapeptide repeat family. These proteins protect DNA gyrase and topoisomerase IV against quinolone inhibition. Typically, *qnr* genes are found within *sul1*-type integrons and are often associated with mobilizing or transposable elements within plasmids (Lee et al., 2021). The next defensive mechanism is acetylation of quinolones. The *aac(6′)-Ib-cr* gene encodes an enzyme known as aminoglycoside acetyltransferase, which has the ability to confer resistance simultaneously against both aminoglycoside antibiotics and quinolones/fluoroquinolone antibiotics (Lee et al., 2021; Gharbi et al., 2023). In addition to *aac(6′)-Ib-cr* gene, the newly described plasmid-mediated phosphorylase gene (*crpP*) was responsible for inactivating ciprofloxacin with the reaction of aminoglycoside phosphotransferase. This gene was detected in pUM505 plasmid, isolated from a clinical *Pseudomonas aeruginosa* isolate (Chávez-Jacobo et al., 2018; Ruiz, 2019; Lee et al., 2021). However, additional research is required to confirm the presence of *crpP* gene in the *Salmonella* genome. The third resistance mechanism is the depletion of the intracellular concentration of quinolones via efflux pumps. Plasmid genes can also lead to resistance by promoting the production of drug efflux systems, such as *QepAB* and *OqxAB*. These efflux pumps help remove quinolones from the bacterial cell, thereby enhancing quinolone and fluoroquinolone resistance (Jacoby et al., 2014; Lee et al., 2021). These resistance mechanisms collaboratively contribute to the challenge of combating quinolone resistance in bacterial infections.

A study conducted in Brazil aimed to investigate the occurrence of PMQR in *Salmonella* and its correlation with susceptibility to fluoroquinolones. The study collected a total of 129 samples from diverse sources, including animal-derived food, environmental samples, animals, and humans. Among the isolated samples, *S*. Typhimurium and *S*. Enteritidis were identified. Interestingly, these isolates displayed resistance to a range of fluoroquinolones, including enrofloxacin, ciprofloxacin, ofloxacin, levofloxacin, and nalidixic acid. Among the isolates, *qnr* genes were detected in 15 instances, with 8 carrying *qnrS*, 6 carrying *qnrB*, and 1 carrying *qnrD*. Additionally, the *aac(6′)-Ib* gene was identified in 23 isolates. Furthermore, the integron gene was identified in 67 of the isolates (Pribul et al., 2017).

A recent study examined the potential presence of PMQR genes within *Salmonella enterica* (Lee et al., 2021). The study involved collecting *Salmonella enterica* samples from patients diagnosed with salmonellosis between 2016 to 2019 in South Korea. Among the 208 clinical isolates of *Salmonella* obtained from humans, thirty-four strains exhibited reduced susceptibility to fluoroquinolones. Within this subset, 22 *Salmonella* strains were found to carry single PMQR genes, including *qnrA*, *qnrB*, or *qnrS*, while three *Salmonella* strains were observed to harbor two PMQR genes, specifically *qnrS* and *aac(6’)-Ib-cr*, or *qnrA* and *qnrB* (Lee et al., 2021).

## 7 Creation of biofilm barriers

Bacterial biofilms can be described as clusters of bacteria that stick to a surface and are enclosed by a self-produced matrix (Vestby et al., 2020). *Salmonella* biofilm extracellular matrix is mainly composed of cellulose, biofilm-associated protein, O-antigen capsule, curli protein (amyloid fimbriae), and extracellular DNA (Daxin, 2016). In *Salmonella*, curli proteins are thin hair-like filaments encoded by *csgBAC* and *csgDEFG* operons. The primary roles of curli proteins include facilitating surface adhesion, promoting cell aggregation, and formation of biofilm. Additionally, curli proteins are involved in mediating the adhesion and invasion of host cells, and they are also effective in activating the host’s inflammatory response (Steenackers et al., 2012; Baugh, 2014; Daxin, 2016). In *Salmonella* biofilms, cellulose, a key exopolysaccharide made up of β-1-4-D-glucose units, is synthesized by two operons, *bcsABZD* and *bcsEFG* (Daxin, 2016).

The formation of biofilms in *Salmonella* increases its chances of survival in hostile environments and facilitates resistance against antimicrobial compounds (Trampari et al., 2021). The formation of *Salmonella* biofilms facilitates contributing to its persistence in particular surface areas (Sakarikou et al., 2020). These biofilms can develop on both living and non-living surfaces (Daxin, 2016). For instance, *Salmonella* biofilms can be found on biotic surfaces such as epithelial cells within a host, including human cholesterol gallstones, and the chicken intestinal epithelium (Castelijn, 2013). *Salmonella* biofilms facilitate *Salmonella* to survive in several conditions such as elevated temperatures, malnourishment conditions, acidic pH, diverse atmospheres, and antimicrobials (Ćwiek et al., 2019). Moreover, bacterial biofilms play an important role in antimicrobial agents through a variety of mechanisms (Cadena et al., 2019). Biofilm production is recognized as one of the best AR mechanisms. Several mechanisms of biofilm structure contribute to antimicrobial resistance. The activation of efflux pumps, limited penetration of antibiotics through the biofilm polysaccharide matrix, quorum-sensing, physiological alterations due to slow growth rates and starvation process, and persisted bacterial cells-like mechanisms allowed biofilms to resist the antimicrobials (Andersen et al., 2015). Furthermore, bacterial cells in biofilms exhibit greater AR than planktonic cells due to their several mechanisms (Ćwiek et al., 2019). Moreover, *Salmonella* forms biofilms on food products, which cause foodborne illnesses, and are challenging to eliminate due to their higher resistance to antibiotics compared to planktonic cells (Alenazy, 2022a). Both typhoid and non-typhoid *Salmonella* were able to perform biofilms. Usually, non-typhoidal *Salmonella* biofilms cause problems in several industries such as veterinary and medical settings. As well as biofilms of *S.* Typhi can be identified on gallstones during infection (Baugh, 2014). The formation of biofilms is important to *Salmonell*a for their growth and survival in the environment. *Salmonella* covered by biofilm exhibits increased resistance to chemical agents such as antibiotics and disinfectants, as well as to physical stresses. Also, the creation of biofilms enhanced *Salmonella* virulence (Daxin, 2016). *Salmonella* biofilms are resistant to several antibiotics such as ampicillin, ciprofloxacin, gentamicin, tetracycline, or third-generation cephalosporins such as ceftriaxone and cefotaxime (Ćwiek et al., 2019).

One of the key mechanisms through which *Salmonella* biofilms develop AR is efflux. Among the multi-drug efflux pumps in *Salmonella*, the AcrAB–TolC efflux system is well-characterized. The MDR efflux pumps found in *Salmonella* are classified into four distinct drug efflux families: five in the RND family, two in the MFS family, one in the MATE family, and one in the ABC family. The inactivation of any multi-drug efflux pumps reduces biofilm production in *Salmonella* Typhimurium (Baugh, 2014). Additionally, the presence of mutants in *S.* Typhimurium, such as *tolC*, *acrB*, *acrD*, *acrEF*, *mdtABC*, *mdsABC*, *emrAB*, *mdfA*, *mdtK*, and *macAB*, can hinder biofilm formation because *Salmonella* strains possessing these mutants lack MDR efflux systems (Baugh, 2014; Daxin, 2016). Nonetheless, a study reported that changing efflux function decreases the formation of *Salmonella* biofilms (Trampari et al., 2021). The AcrD efflux pump is important to *Salmonella* biofilm formation. Mutations in AcrD significantly reduce both biofilm formation and the expression of key biofilm proteins encoded by *csgBD* (Buckner et al., 2016). In *S.* Typhimurium, *tolC* and *acrB* mutants cannot form a functional biofilm and suppress the transcription of both curli operons (*csgBAC* and *csgDEFG*) (Baugh, 2014).

The extracellular matrix within microbial biofilms serves a critical function, such as providing structural support, facilitating communication between cells, and protecting the bacterial community by blocking antibiotics from penetrating the bacteria (Vestby et al., 2020; Singh et al., 2022; Prinzi and Rohde, 2023). *Salmonella* biofilms enhance the likelihood of persistence in hostile environments by facilitating defiance against antimicrobial compounds. This occurs because of their structure and the composition of their extracellular matrix (Trampari et al., 2021). A study demonstrated that *Salmonella* biofilms show resistance to ciprofloxacin due to the formation of biofilm structure (González et al., 2018). In addition, *Salmonella* exhibits tolerance to triclosan, a widely used biocide against bacteria, including *Salmonella* (Tabak et al., 2007). This tolerance is attributed to the limited diffusion of triclosan through the extracellular matrix formed by *Salmonella* as part of its biofilm formation. Moreover, extracellular DNA (eDNA) within *Salmonella* biofilms enhances AR to specific antibiotics by altering the outer membrane’s magnesium ion concentration. DNA, being an anionic molecule, can chelate cations like Mg^2+^. Notably, Mg^2+^ restriction in *S.* Typhimurium acts as an environmental signal, triggering the induction of the PhoPQ and PmrAB two-component systems, which subsequently promote AR mechanisms (Dincer et al., 2020).

## 8 Conclusion and future perspectives

In conclusion, this review highlights the complex nature of AR in *Salmonella*, emphasizing the various resistance mechanisms that include drug efflux, target site modification, permeability alterations, and biofilm formation. The extensive and improper use of antibiotics in both clinical and agricultural settings has considerably contributed to the acceleration of resistance traits among various *Salmonella* serovars. The rising MDR *Salmonella* presents an alarming public health challenge with significant implications for treatment efficacy and infection control.

Efforts to combat this issue must be multi-pronged, emphasizing the development of novel antimicrobial agents, such as neoglycosides (Labby and Garneau-Tsodikova, 2013), and the use of antibiotic adjuvants (Wright, 2016). Strategies should also include the combination of multiple antibiotics (Parmanik et al., 2022; Wang et al., 2022a; Lobertti et al., 2024), the creation of enzyme specific inhibitors such as novel β-lactamase inhibitors, AEM inhibitors to overcome aminoglycoside resistance (Wang et al., 2022a), and chloramphenicol acetyltransferase inhibitors to combat chloramphenicol resistance. Moreover, efflux pump inhibitors, including berberine and palmatine (Morita et al., 2016; Aghayan et al., 2017; Li and Ge, 2023), should be employed. The use of nanoparticles as drug delivery systems and the incorporation of plant-derived metabolites (Parmanik et al., 2022) are promising approaches. Furthermore, the judicious use of existing antibiotics, enhanced surveillance for resistance patterns, and rigorous infection prevention strategies are essential components of this comprehensive approach.

Additionally, public health education on antibiotic stewardship and the importance of comprehensive hygiene practices in food preparation are critical in curbing the spread of these resistant pathogens. Future research should focus on innovative approaches to overcome bacterial defensive mechanisms, such as the development of new therapeutic molecules, the use of bacteriophage therapy, the use of competitive exclusion to suppress pathogenic bacteria in the food environment, genetic modification of surrogate bacteria using gene editing tools such as CRISPR-Cas techniques to reduce AR *Salmonella* and the exploration of vaccine potentials. To combat the threat of AR *Salmonella* and ensure proper treatment for those infected, coordinated and collaborative actions across the globe are essential.
